# Stories that bridge us: A mixed methods study to understand the impact of a hospital-wide storytelling event

**DOI:** 10.1371/journal.pone.0327384

**Published:** 2025-06-27

**Authors:** Maria F. Nardell, Malini M. Gandhi, Barbara Sarnoff Lee, Amy Wasserman

**Affiliations:** 1 Medicine, Beth Israel Deaconess Medical Center, Boston, Massachusetts, United States of America; 2 Medicine, Brigham and Women’s Hospital, Boston, Massachusetts, United States of America; 3 Harvard Medical School, Boston, Massachusetts, United States of America; 4 Social Work and Patient Family Engagement, Beth Israel Deaconess Medical Center, Boston, Massachusetts, United States; 5 Organizational Development, Beth Israel Deaconess Medical Center, Boston Massachusetts, United States; Western University Faculty of Science, CANADA

## Abstract

Oral storytelling events for healthcare professionals are gaining in popularity, yet evaluation of these initiatives is scarce. We designed and assessed the impact of a hospital-wide storytelling event at an academic medical center in New England. This study was grounded in social constructivism, which posits that knowledge and collaborative meaning-making are socially constructed through interpersonal interactions and shared language. Stories were solicited from interdisciplinary staff on a theme, and six selected storytellers were paired with coaches. The hybrid in-person/virtual event was held in 2021. Attendees were invited to complete a post-event survey, as well as a semi-structured interview or written response. Storytellers were invited to reflect via a post-event focus group or written responses. Qualitative data were coded using a mixed inductive and deductive content analytic approach. Survey data were analyzed using descriptive statistics. The storytellers included representation from internal and emergency medicine, nursing, infrastructure project management, and research administration. The 155 attendees included 25 in-person/130 virtual. Qualitative data (nine participants) revealed that sharing stories fostered interpersonal connection and a sense of common humanity, enhanced by the storytellers’ vulnerability and diversity. Storytellers valued coaches’ emotional and creative support in co-creating stories with them. Lastly, the event was felt to strengthen the hospital community. These themes were echoed in the survey data (30 participants): > 75% of respondents indicated that the event helped them reflect on their values, connect with others, and access a sense of purpose. A multidisciplinary hospital-wide oral storytelling event is one way to enhance self-reflection, interpersonal connection, and a sense of community among healthcare professionals.

## Introduction

Medicine is rooted in storytelling. The relationship between patient and healthcare provider is fundamentally an act of sharing and honoring human stories and life experiences [1]. The past several decades have seen the growth of narrative medicine, which aims to enhance physicians’ relationships with patients, colleagues, and society [2]. Through engaging with storytelling and literature, “narrative competence” promotes empathy, reflective capacity, and communication skills central to caring for patients [2–5]. It also promotes the wellbeing of healthcare providers [4,6].

Building on “Story Slam” events such as *The Moth* [7], oral storytelling events for healthcare providers have emerged as a medium for members of the medical community to give voice to and reflect on their own professional and personal stories. The Nocturnists, a San Francisco-based podcast in which healthcare providers share their stories “from the world of medicine,” has proven resonant within the healthcare community [8], and similar initiatives have been established elsewhere [4,9,10]. In addition to building narrative competence and storytelling skills, oral storytelling events for healthcare professionals and students humanize physicians, build community within the medical community, and foster resilience among providers [4,8,9,11,12]. For example, a “Story Slam” for medical faculty and trainees at the University of Minnesota was found to promote connection among participants, restore a sense of meaning in their work as physicians, generate hope, and counter burn-out [4]. Stanford’s Medicine & the Muse program organizes personal storytelling workshops for medical students, allowing for community-building among peers [13]. A study of an oral storytelling event at the University of Massachusetts Medical School reported benefits for participants in combatting emotional exhaustion and depersonalization as well as promoting professional development [11].

The challenging years of the Covid-19 pandemic raised awareness of the need to cultivate the wellbeing of physicians, nurses, and other clinical staff [14]. Moreover, data on rates of burnout – characterized by exhaustion, negativity toward one’s job, and reduced professional efficacy [15] – among healthcare workers across 206 large healthcare organizations in 2020 showed that while the burnout rate for clinical staff ranged from 47.3–56.0%, nearly half (45.6%) of non-clinical staff also experienced burnout [14]. Thus, it is critical for both providers and other hospital staff to have strategies to process grief and loss and to build connection and community. For example, in a virtual storytelling event for radiology students, trainees or faculty nationwide, participants shared personal stories of their experiences during the COVID-19 pandemic, which was found to improve their sense of emotional and professional growth [12].

Despite the benefits of storytelling in promoting emotional and professional growth and in fostering connection among colleagues, the incorporation of oral storytelling by providers into medical settings is still limited in most organizations, and research on its impact and scope remains scarce. Most storytelling events focus on stories explicitly related to medicine [9,11,16,17], even though healthcare providers’ non-professional lives also inform their professional practice. There is little research on the degree to which healthcare providers’ stories focus on personal or professional experiences influence outcomes for both storytelling and audience participants. In addition, most narrative medicine and storytelling programs are siloed within individual healthcare professions (e.g., medicine, nursing) [4,5,18,19] or departments (e.g., oncology, radiology) [12,16]. Fewer initiatives have envisioned storytelling at an institution-wide level to bring together storytellers and attendees from across different roles in healthcare, from administration to clinical care to research to other support staff [20]. It has not been well-studied how the inclusion of an interdisciplinary range of healthcare-based staff as storytellers may influence storytelling programs and events.

Given the benefits of storytelling and a desire to bolster a sense of community within our medical center during the Covid-19 pandemic, we designed a hospital-wide storytelling event at the Beth Israel Deaconess Medical Center (BIDMC) in Boston (“BIDMC Stories”) in December 2021. The event featured stories from six members of the BIDMC community, all centered on the theme “Defining moments: the stories that shape us.” Several notable elements of our storytelling event allowed exploration and analysis of novel questions about this medium. We invited story submissions from staff in any role within the hospital, allowing for exploration of storytelling events as a way of fostering interprofessional community. We hoped this diversity of roles would allow for exploration of storytelling events as a way of fostering interprofessional community, building on literature describing the positive impact of communication about personal, emotional elements of patient care among multi-disciplinary teams of healthcare professionals in promoting connection and teamwork [21]. Additionally, unlike medical storytelling events in which stories are linked directly to medicine, our event welcomed stories that encompassed both medical and non-medical facets of storytellers’ lives. We sought to explore how the process of developing and sharing human narratives through a storytelling event, regardless of explicit connection to medicine, can generate community, resilience, and empathy in the medical community.

We conducted a mixed methods study of the BIDMC Stories event to understand how these choices impacted the experience of our event for our audience and storyteller participants, and more broadly, for storytelling initiatives within medical settings. Our qualitative data included audience comments during the event, individual interviews with audience members, and a focus group with storytellers. In addition, we asked audience members to complete a post-event survey to quantitatively assess the event’s impact in areas such as interpersonal connection, self-reflection, and their interest in future storytelling events.

### Theoretical framework

We used social constructivism as a theoretical framework to help interpret our qualitative and quantitative data. Social constructivism theories posit that people’s understandings of the world are produced by interactions between individuals in society, vary by context, and impact people’s behaviors [22]. Social constructivism originated in the fields of sociology and anthropology, rooted in the works of theorists including Dewey, Piaget, and Vygotsky [23]. Social constructivism has been used in medical education to understand learning as a social process [24]. It has also been applied as a framework for understanding how oral stories shared among health providers can lead to learning and the construction of meaning [4]. Humans engage in a process of personal reflection to construct meaning and personal identity from our social interactions, environment, and culture. In preparing and sharing stories with others in their professional community, storytellers are able to test their constructs and continually refine their meaning of experiences, while listeners are encouraged to reflect on and construct meaning from their own stories [25]. Language has a multi-faceted role within social constructivism; it serves as a tool for communication with others (i.e., the literal words used to tell stories) and also creates common knowledge, constructs shared reality, and carries the values, norms and beliefs of a cultural context [26]. In an era in which there is increasing recognition of the need to address system-based, root causes of healthcare provider distress [27], this collaborative process also lends itself toward the development of shared language to give voice to underlying systemic problems and possible solutions to enhance provider wellbeing.

## Methods

### Event planning

The storytelling event was planned by an interdisciplinary team of professionals at our hospital. The Steering Committee included representation from medicine, human resources, social work, nursing, administration, communications, and media services. The theme for the event – “Defining moments: the stories that shape us” – was selected to elicit a broad range of stories related to or unrelated to healthcare. A call was put out to all staff, trainees, and students at BIDMC for submissions of a one-hundred-word synopsis of a story on this theme. We advertised through posters, flyers, staff and trainee list-serves, social media, and word-of-mouth. All advertising was in English given that most healthcare staff at our institution speak at least some English. However, there were no language restrictions for submitting a story synopsis. Interpreters were available to translate any synopses not in spoken English, and to translate during the live event as well, if needed.

A Story Selection Committee, including representation from medicine, nursing, administration, and human resources, convened to review all story submissions. We had a target of six or seven stories for the event so as to allow for each story to be 6–8 minutes and for the entire event to last one hour. Submissions were rated on the basis of three main criteria: 1) the story has a clear and engaging arc; 2) the story has a meaningful message; and 3) the story is unique and/or relatable. We selected these criteria based on data showing how stories with these components most resonate with listeners in a healthcare provider community [4]. In addition, we sought to have diversity of representation across of our hospital, including diversity in ethnic or racial background, gender, professional role, and professional experience level. We also sought to have diversity of the types of stories shared, including both emotionally heavy and light-hearted stories. Of the 23 submissions received, 12 potential storytellers were invited to meet virtually with the Story Selection Committee to provide more detail on their story. Ultimately, seven storytellers were selected to share their stories, and six storytellers elected to proceed with the event. The event was held on a weekday evening in December 2021. Given Covid-19 restrictions, the event was held in a hybrid virtual-in person format, in which storytellers presented their stories in person in a hospital conference room to a small in-person audience of fellow storytellers and event organizers. Two senior hospital leaders introduced and closed the event, respectively. In between, the six storytellers were each introduced and then presented their stories, with a two-minute pause after each story to allow for audience reflection. The hospital media team livestreamed the event over Zoom for all other audience members, and it was recorded for later viewing.

### Story development

The six storytellers were each paired with one of two professional story coaches with prior experience coaching healthcare staff on developing and presenting stories for live storytelling events. The coaches met individually with each of their three storytellers between three and five times (depending on the storytellers’ availability and needs) during the two months leading up to the event. The coaches helped participants craft and present their story by focusing on the most important components, eliciting sensory and emotional details, and using their physical presence to engage listeners. Storytellers were asked to prepare their stories such that they could deliver it live without notes.

### Data collection

The recruitment period for this study occurred from December 2^nd^ to December 22^nd^, 2021. During the event on December 2^nd^, we invited virtual attendees to share their reflections in the group Zoom chat. At the close of the event, we invited attendees to complete a brief ten-item, Outlook-based survey about their experience of the event, which included both Likert-based and open responses. (See Appendix.) The post-event survey was designed to understand attendees’ experience of the event and assess the likelihood of their participation as either an attendee or storyteller in the future. A QR code for the survey was posted on Zoom at the end of the event and emailed to all Zoom participants the day after the event. The final question of the survey asked participants if they would be willing to participate in a qualitative interview to share more in-depth reflections about their experience of the event. Those participants who responded yes to this question were contacted by MFN by email. The semi-structured qualitative interview guide (see Appendix) was created to understand how storytelling events may impact healthcare communities, probing for themes including community and connection, diversity of professional roles, burnout, and personal reflection on one’s own stories. To make it more feasible for more people to respond despite their busy schedules, participants were given the option to submit written responses, an audio or video recording, or schedule an interview. Responses to the survey and in-depth interview questions were collected over the course of the subsequent three weeks, de-identified, transcribed when necessary, and maintained in a secure database for analysis.

In addition, we asked for volunteers among the six storytellers to share their experience of the event through either a focus group or written reflection. The in-depth focus group guide (see Appendix) focused on the storytellers’ motivations for participating in the event and sharing their story, their experience preparing their story, their experience delivering their own story and hearing the stories of others, and how they believe that storytelling events may impact hospital communities. This focus group was held two and a half weeks after the event, and the audio recording was transcribed and stored securely.

For the survey and in-depth written responses, written information about the study was provided on the webpage (survey) or in the email (written responses), including information about informed consent and the contact information for the PI (Nardell) and BIDMC Human Subject Protection Office. For the oral interviews and focus group, which were conducted by the PI, the PI verbally reviewed this information with each participant to obtain verbal informed consent before the start of the interview. This consent process was witnessed and documented by the PI, and it was approved by our ethics committee.

### Data analysis

For each item in the post-event survey, we conducted descriptive statistics to present the percentage of responses for each Likert scale category (“Not at all,” “A little bit,” “Somewhat,” “Quite a bit,” and “Extremely.”) Qualitative responses to the open-ended questions in the survey, as well as the comments in the Zoom chat during the event, were analyzed along with the qualitative data from the individual interviews, focus group, and written responses. Analysis of these qualitative data followed a mixed deductive and inductive approach [28]. Our coding team (MFN, MMG) developed an initial codebook based on key concepts from the interview protocol and emergent codes from a review of the first four interview transcripts, drawing on concepts from social constructivism. The coding team met three times over the course of a month to discuss emerging themes through repeated review of the transcripts and memoing. Through these meetings, we refined the codebook by adding new codes, modifying definitions of existing codes, and merging existing codes. Discrepancies were resolved through discussion, and an audit trail of each codebook version was maintained. Interviews were coded in Dedoose [29]. MFN and MMG both coded four transcripts to ensure consistency until consensus was reached. MMG subsequently completed coding for the remaining transcripts. MFN and MMG then reviewed and summarized the excerpts for each code. Repeated patterns of content formed the basis for broader themes [28].

### Ethical approval

The ethics committee at Beth Israel Deaconess Medical Center reviewed the study and determined that it was exempt (2021P000989). All participants in the qualitative interviews or focus groups provided oral informed consent.

## Results

As shown in Table 1, the six storytellers included two attending physicians (Internal Medicine and Podiatry), a resident physician (Emergency Medicine), a nurse (Gastroenterology), an infrastructure project manager, and a research administrator. Experience levels varied from early career professionals to those who had been in their professional role for over 25 years. Men and women were equally represented. Stories ranged from those that were focused on professional experiences in healthcare to those related to personal illness or experiences unrelated to healthcare (e.g., a defining childhood moment, the moment of meeting one’s spouse.) The six storytellers represented three racial categories (two Black, two Asian, two white).

**Table 1:** Descriptive statistics on storytellers, event attendees, and study data sources.

Storytellers	
Total # of storytellers	6
Gender of storytellers	
Men	3 (50%)
Women	3 (50%)
Race of storytellers	
Black	2 (33.3%)
Asian	2 (33.3%)
White	2 (33.3%)
Storytellers’ role in hospital	
Attending physician	2 (33.3%)
Resident physician	1 (16.7%)
Nurse	1 (16.7%)
Non-clinical staff	2 (33.3%)
Content of stories	
Experiences in healthcare	3 (50%)
Personal experiences unrelated to healthcare	3 (50%)
Event attendees	
Total # of event attendees	155
Mode of attendance	
Zoom	130 (83.9%)
In-person	25 (16.1%)
Study data sources	
Storyteller responses	
Participation in focus group	2 (33.3%)
In-depth written response	1 (16.7%)
Event attendee responses	
Post-event survey response	30 (19.4%)
Post-event in-depth qualitative responses	7 (23.3% of survey respondents)
Written qualitative survey	4
Qualitative interview	3
Zoom chat comment during event^a^	7 (5.4% of Zoom attendees)

^a^Indicates the number of unique Zoom attendees that provided zoom chat comments

There were 25 individuals present in-person for the event, including the six storytellers, the committee members, senior hospital leadership, and the media team. There were 130 attendees who watched the event live on Zoom. Thirty attendees completed the post-event survey, and seven attendees volunteered to share additional reflections through written responses to the qualitative survey (four participants) or through a qualitative interview conducted by MFN (three participants). Two of the six storytellers volunteered to participate in a focus group after the event, and one provided a written response.

### Qualitative data

We identified three major themes from the qualitative data, each drawing on elements of social constructivism. First, co-developing, sharing, and hearing stories allowed both storytellers and listeners to make meaning of their lives, which in turn promoted interpersonal connections with each other. Second, coaching for the storytellers was essential in providing creative and emotional support to collaboratively craft and deliver their stories. Third, the storytelling event helped promote a sense of institutional belonging for attendees and storytellers.

#### Theme 1: Constructing meaning through developing and sharing personal stories leads to interpersonal connection.

As shown with representative quotes in Table 2, storytellers shared how the process of co-constructing their personal stories helped them to construct identity and meaning from transformative experiences in their lives. They emphasized that they “carried [stories] within me,” and thus they appreciated the opportunity for a “creative outlet,” which allowed for better self-understanding of their life history and for “closure.” Attendees described how “it’s always meaningful to get access to someone else’s life story,” which then prompted them to reflect on stories from their own lives. This process of storytellers and attendees engaging in personal reflection within the context of a shared storytelling experience allowed them to recognize parts of their experiences and emotions in the stories of others. It evoked “a sense of comradery and connectedness” to colleagues, as well as a sense of shared humanity, which was enhanced by the diversity of the storytellers. Attendees commented on how “it was great that different disciplines were represented,” and “everyone had someone who looked like them, so that they could identify better.” In addition, an attendee described how “seeing different perspectives…engenders greater empathy, appreciation, and understanding” of others.

**Table 2:** Representative quotations on personal meaning-making and interpersonal connection.

Sub-theme	Quote
Meaning-making from life experiences	“It’s just helping me process a lot of my history with diabetes.” *(Storyteller, focus group)*
	“I found myself thinking about different stories in my life; what has impacted me and made me who I am today.” *(Attendee, in-depth written response)*
Interpersonal connection and sense of shared humanity	“What also surprised me was I realized that I had never (my white privilege) heard the N word spoken – its force and vitriol caused me to shudder. The utterance of the word and the emotional context provided allowed me to understand its impact. The plight of the refugees fleeing Vietnam and not knowing if and when she would ever see her sister again, the sea sickness, the hunger, the fear. It brought the immigrant experience to life in a way that is often overlooked in the endless political debates.” *(Attendee, in-depth interview)*
	“It creates a sense of family in that we’re all in this together. One wants to reach out to those who shared and say, ‘I get it’ or ‘Your story touched a part of me that brought hope’ or ‘Your pain and struggle was so real and I understand that pain in a similar way.’” *(Attendee, in-depth written responses)*

#### Theme 2: Story coaching is essential for creative and emotional support for storytellers.

Storytellers highlighted the importance of their story coach for co-constructing their story over the course of the seven-week timeframe. (See Table 3 for representative quotations.) They described an iterative process in which they presented their material to the coach, and the coach helped them to refine the story by focusing on the most essential elements. This collaborative development process was key in translating “so many complicated events and feelings” or written material into a well-crafted, orally delivered, co-constructed story for a live audience. In addition, coaches provided valuable emotional support and “nurturing” for storytellers by building their confidence and courage to share vulnerable parts of themselves. The storytellers identified that many people “stress out about the [storytelling] process” because of a fear that “there’s no one guiding me through this,” which becomes a “barrier to entry.” One storyteller described not being “a talkative type when I tell my family or friends stories at work,” but working with the coach helped to “explore the other stories at work I could learn to tell.” Attendees commented on the inspiration they felt from the “vulnerability and courage” of the storytellers, though some expressed reticence to share their own stories given the “very high bar” set by the inaugural storytellers.

**Table 3:** Representative quotations on the value of coaching in providing creative and emotional support.

Sub-theme	Quote
Creative support	“I went in with an expectation of what I wanted to write…there were a lot more factors I was thinking about in terms of setting the scene, the context. And then [my coach] was like, No, you got to cut all that out. Like, it’s really just about you, and what matters to you…So I thought that was really effective, because it really helped me focus on what I needed.’” *(Storyteller, focus group)*
	“[I was] surprised by how well the stories were written.” *(Attendee, survey)*
Emotional support	“I think the coaching was super valuable, knowing that you have someone who is going to read and give you objective, dispassionate feedback on how you’re doing. That was great and nurturing too…Like, I think that is a big fear - that there’s no one really guiding me through this, there’s no one to know if I’m editing it correctly…And so I think the coaches is very high yield.” *(Storyteller, focus group)*
	“The storytellers had a lot of courage to get up in front of so many people and talk about something so personal.” *(Attendee, survey)*

#### Theme 3: A culture of storytelling can help promote a sense of institutional belonging.

In addition to building interpersonal connection, our data revealed that the storytelling event fostered connection to shared values within the hospital institution, as shown with representative quotations in Table 4. Both storytellers and attendees articulated how storytelling can help build “a strong sense of belonging and feeling valued.” They further described how making the event “an institutional thing” would signal to others that “we care about storytelling and humanism.” Participants gave numerous suggestions for ways to build upon an organizational culture of storytelling. For example, they suggested sharing event recordings broadly (e.g., with new hires, on a podcast), creating a venue to share “mini-stories,” starting patient rounds, conferences, and other meetings with short patient or provider stories or poetry, and collaborating with other humanities initiatives at the hospital (e.g., printing stories from our event in the hospital’s literary magazine.) Participants also had suggestions for ways to improve future storytelling events, including further diversifying the storytellers by encouraging employee participation from areas such as environmental and food services.

**Table 4:** Representative quotations on how a culture of storytelling can help to promote a sense of institutional belonging.

Sub-theme	Quote
Impact of event in strengthening institutional community	“When I was listening to the stories, I kept thinking, what a wonderful place to work which invites us to a storytelling event.” *(Attendee, in-depth written responses)*
	“Making this [event] an institutional thing here at BI would...really drive home the image that we care about storytelling and humanism in our patient care.” *(Storyteller, focus group)*
Other methods of further promoting a broader institutional culture of storytelling	“Even on rounds, walk rounds, if one started with - you’ve got a whole team, and if you said, what was the best moment you had with a patient in the last 24 hours? Somebody’s going to tell you a vignette and a story. It takes three seconds.” *(Attendee, in-depth interview)*
	“My fourth-year senior project [in medical school] was revolving around 55-word stories… we had like a small workshop where you just write 55-word stories…I think it’s a really easy and great avenue. And it’s very, very powerful in terms of the literature around it, especially in the medical community.” *(Storyteller, focus group)*

### Survey data

**Fig 1:**
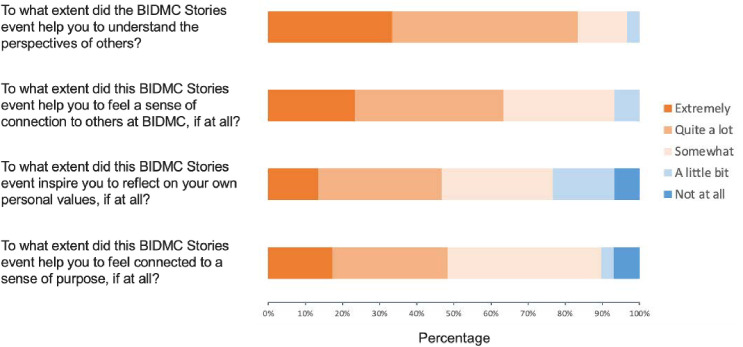
Interpersonal connection and self-reflection. The figure below shows survey responses from attendees (N = 30) following the event on the themes of interpersonal connection and self-reflection.

**Fig 2:**
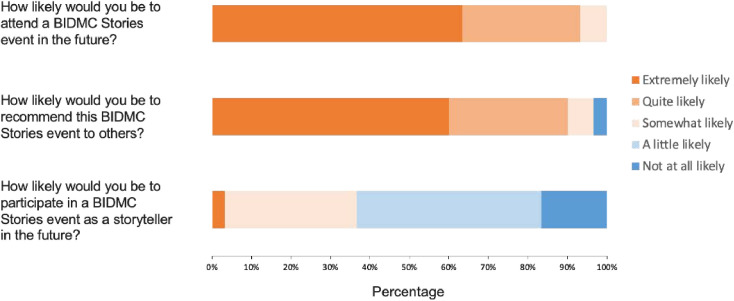
Future participation in storytelling events. The figure below shows survey responses from attendees (N = 30) following the event on questions related to anticipated attendance and participation in future storytelling events.

The survey data echoed many of the themes found in the qualitative data. As shown in Fig 1, more than 80% of attendees felt that the event helped them “quite a lot” or “extremely” to understand the perspectives of others, and over 60% reported feeling “quite a lot” or “extremely” a sense of connection to others within the institution. Nearly half responded that they event inspired them “quite a lot” or “extremely” to reflect on their own values and feel a sense of purpose. As shown in Fig 2, the vast majority (>90%) of attendees would attend a future storytelling event and recommend it to others. However, only 36.7% reported being at least “somewhat likely” to participate as a storyteller in the future.

## Discussion

This mixed methods study of a hospital-wide storytelling event highlighted its impact in helping storytellers and audience members to co-construct shared meaning through personal reflection. Reflective practices for clinical caregivers have been found to enhance medical education, professional development, and well-being in a number of ways, including skills development (e.g., to take patient histories [30]), problem-solving abilities (e.g., to achieve more work-life balance [31]), professional and relational awareness (e.g., to understand interdisciplinary team roles [32] and social determinants of health [30]), and empathetic attitudes towards one’s own and other’s emotions (e.g., to work with patients and team members [33]). [34] Social constructivism suggests that these and other benefits are further enhanced by the shared construction of meaning through working with story coaches as well as the interactive experience of a storytelling event. Working with a coach as well as hearing and reflecting on the perspectives of others allows for further refinement of one’s own knowledge and beliefs across both professional and personal realms [35,36]. Participants didn’t distinguish between the meaning that they derived from healthcare-related and other personal stories; however, the stories that audience members mentioned the most were those in which the storytellers conveyed deep emotions and/or a personal struggle. Used well, vulnerability may be a strength that allows listeners to relate to the emotions of storytellers more fully, and it may also help storytellers to make more sense of their own experiences [37].

We found that participants felt a sense of interpersonal connection and shared humanity through the event. These themes have been found in other oral storytelling initiatives as well [4,11,38], and they may promote wellness through a sense of belonging and support [39]. The high levels of emotional exhaustion and depersonalization reported by healthcare workers during and after the Covid-19 pandemic were not new [40], though the pandemic exacerbated these challenges and made them more visible and urgent to address [41–43]. Proactively providing opportunities to improve the wellness of the healthcare workforce, including building interpersonal connection, is both a moral and public health priority [44]. We also found that participants appreciated the diversity of storytellers, including representation from a range of hospital teams. Research shows that strong interprofessional healthcare collaboration may improve healthcare providers’ wellbeing and also improve patient outcomes [45]. Bringing together a diversity of hospital team members for storytelling events such as this one may inspire future interdisciplinary relationship-building. Moreover, in addition to oral stories as a medium, other forms of creative expression have been used within various hospital institutions to promote reflection and connection, including writing, art, dance, film and music [46,47]; this variety of approaches may appeal to a range of people with different interests and experiences.

Crafting narrative is a creative skill, and not everyone starts with the same level of proficiency. Our study revealed that coaching is an important piece of deepening storytellers’ reflective practice skills and translating personal reflections into a cohesive, co-constructed story that will engage a diverse audience. Coaching also provided essential emotional support to our storyteller participants, none of whom had delivered a story to a live audience before. Other medical storytelling events also provide coaching to storytellers [4,8], including a coaching workshop for medical professionals [48], though coaching storytelling within healthcare is limited as compared to fields such as executive leadership development [49]. Social constructivism suggests that the interactions between the coach and storyteller is crucial, not only for technical guidance on story crafting (e.g., cutting extraneous material), but also for co-creating new meaning from stories which may not have otherwise emerged through self-reflection alone. Research in the field of leadership development has found that the effectiveness of coaching is related in large part to the strength of the relationship between coach and coachee, for which unconditional support and acceptance are key [50]. Similarly, we found that storytellers’ perception of the nurturing support they received from their coach allowed them to feel more comfortable to explore meaning and share vulnerably. While not all storytelling initiatives may have access to or be able to provide one-on-one professional coaching, peers may be trained to be peer coaches for each other as well. This approach has been used in the business world [51], and it may allow for broader use of storytelling initiatives in healthcare as well.

**Fig 3:**
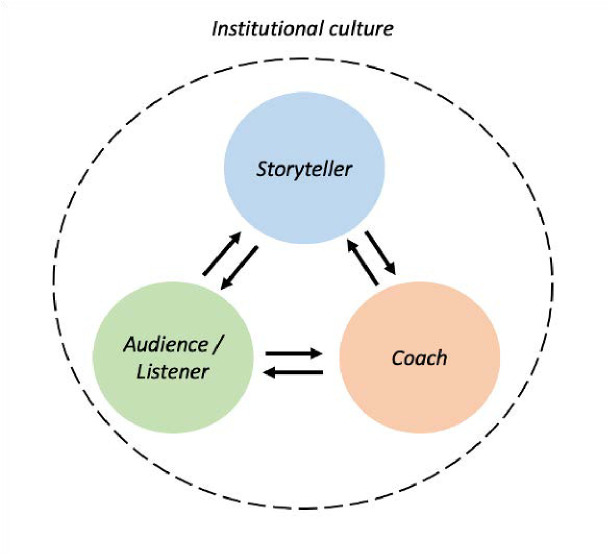
Social constructivism as a framework for meaning-making through a storytelling event.

Our third major finding relates to the importance of storytelling as a tool for growing a stronger community culture. Participants expressed that the storytelling event fostered feelings of pride and belonging in BIDMC as an institution, and that the event embodied a sense of shared institutional values. Our participants voiced strong support for future events and suggested numerous ideas for further embedding storytelling initiatives into the hospital to create a broader institutional culture of storytelling. Many of our participants’ ideas are supported by similar experiences in other medical settings, including the use of 55-word stories for personal reflection and teaching [52], development of podcasts to capture stories from the medical community [8], and incorporation of storytelling or personal reflection on creative pieces into hospital rounds [53]. Notably, healthcare organizations are struggling to retain employees [54], and on top of traditional strategies focused on wages and other benefits, a “purposeful culture” has been found to be a key component of organizational satisifaction [55]. A strong sense of humanism within the hospital may also attract medical students who value its role within their medical training [56]. This ability of storytelling to not simply promote connection between the storyteller and listener but also to generate a broader sense of community is supported by social constructivism, which posits that language not only links individuals but also serves to build shared reality and construct the values and norms of a community. Through this lens, storytelling can be a means for both individual and systemic change, offering shared language that can help give voice to and address larger root causes of provider distress. Fig 3 summarizes social constructivism as a framework for the role of storytelling, depicting the interlocking, multi-directional exchange of language and meaning-making between storyteller, coach, and listener, which together form a wider institutional culture.

In addition to their impact on the hospital community more broadly, oral storytelling events may hold particular value for both undergraduate and graduate medical education. Several stories told at our event were often directly related to clinical work or relayed personal anecdotes relevant to the speaker’s clinical experiences; for students and residents, these stories may be all the more visceral and resonant given the novelty of the clinical space for trainees and the intensity of their work hours. A growing body of literature suggests that implementation of narrative medicine initiatives in medical education can help students and trainees reflect on and process this novelty and intensity, while also helping to support their empathy for patients and connection to each other [16,32,57–61]. Developing narrative and reflective skills early in medical training could help build increased resilience later on, guarding against future burn-out. Evaluation of the use of oral storytelling events specifically in the context of medical education warrants future study.

Limitations of this study include the focus on a single event at one institution and small sample sizes, which limits generalizability. It is possible that there was a self-selection bias among storytellers and attendees who chose to participate in our study. Nonetheless, our findings suggest that storytelling is one modality that benefits at least a subset of healthcare professionals, and it may considered among a suite of strategies that aim to promote healthcare staff reflection, connection, and community. In addition, future research should evaluate longer-term outcomes of oral storytelling events to assess the ability of these effects to persist, including in medical education or medical research spaces [62], or with novel variations, such as a blend of patient and provider stories. In addition, stories were envisioned as spoken or signed for a live audience, which had the potential to inadvertently exclude staff members unable to communicate in this manner. For future events, we would consider expanding our scope to include other forms of creative expression, such as movement, visual art, or weaving stories into a film. Moreover, while we were prepared to receive non-English story synopses and interpret at the live event for non-English storytellers, if needed, our recruitment of staff members for whom English was not a native language may have been limited by advertising only in English. In the future, we would advertise in other commonly spoken languages among staff in our hospital, such as Spanish and Haitian Creole. There is little guidance in the literature on how others have addressed this issue in medical storytelling initiatives [63], highlighting the need for more awareness about inclusivity in such events. Lastly, while this study focused on how the process of co-developing and sharing human narratives through a storytelling event could contribute to positive benefits within the hospital community, a future project could also consider narrative analysis to uncover common themes within the stories themselves.

Ultimately, our event demonstrates that a multi-disciplinary hospital-wide oral storytelling event can be one tool for enhancing self-reflection, interpersonal connection, and contributing to a sense of hospital community.

## Supporting information

S1 File Survey, qualitative interview guide, focus group interview guide. (DOCX)

S2 Table Survey study data. (XLSX)

S3 Table Qualitative study data. (XLSX)
