# Supplementary material for: Stories that bridge us: A mixed methods study to understand the impact of a hospital-wide storytelling event
Source: PLoS One. 2025 Jun 27;20(6):e0327384. doi: 10.1371/journal.pone.0327384 (PMC12204519; doi:10.1371/journal.pone.0327384)
Supplement: S1 File — (DOCX) [file pone.0327384.s001.docx]

**S1 File. Survey, qualitative interview guide, focus group interview guide**

**1. Post-event survey for attendees**

1. What is your primary role at BIDMC?

- ________ [fill in the blank]
- I am not part of BIDMC

1. Please describe your experience at tonight’s event (e.g. How did you feel? What surprised you? What was meaningful for you?) [text box]
2. One goal of this BIDMC Stories event was to help create a sense of community and connection at BIDMC. Did the event accomplish that goal?

- Not at all
- A little bit
- Moderately well
- Quite well
- Extremely well

1. Another goal of this BIDMC Stories event was to help inspire personal reflection among those who attended. Did the event accomplish that goal?

- Not at all
- A little bit
- Moderately well
- Quite well
- Extremely well

1. How likely would you be to recommend this BIDMC Stories event to friends and colleagues?

- Not at all likely
- A little likely
- Moderately likely
- Quite likely
- Extremely likely

1. How likely would you be to participate in a BIDMC Stories event as a storyteller in the future?

- Not at all likely
- A little likely
- Moderately likely
- Quite likely
- Extremely likely

1. As part of a research project on the use of storytelling events in healthcare communities, we are looking for people to participate in a brief interview about their experience at this BIDMC Stories event. Would you be willing to volunteer? Yes or no. [If yes, skip logic to get name & email.]
2. Any other comments or suggestions for future BIDMC Stories events? [text box]

**2. Semi-structured interview questions for attendees**

- What is your role at BIDMC?
- Do you think storytelling events like BIDMC Stories could help build a sense of community and connection at BIDMC? If so, how? What would help this sense of community and connection to persist?
- What was it like to hear stories from people in different roles across BIDMC? How does it impact how you may think about people who you come across in the hospital?
- We know that it has been a challenging time for people who work in healthcare. Do you think storytelling events can help to reduce feelings of burnout? If so, how?
- After the BIDMC Stories event, have you been reflecting on any stories from your own experiences? If so, why? Have your perspectives on any of these stories changed after attending this event?
- How likely would you be to share a story (with the help of a storytelling coach) at a storytelling event? Are there other ways you would prefer to share your stories? Please explain.

**3. Focus group guide for storytellers**

- First, we are curious about what motivated you to participate in this event.
  - Had you previously participated in storytelling events?
    - If yes, why did you choose to do this one?
    - If no, what inspired you this time?
  - How did you choose to share your particular story?
- What was it like for you to prepare your story?
  - Did your story evolve over time? If so, did you learn new things about yourself or others?
  - Did you feel supported in the process of preparing your story? What else could we have done to improve this process for you?
- What was it like for you to deliver your story to an audience?
  - What surprised you?
  - Would you do it again?
  - Is there anything we could have done to help you feel better prepared?
- What was it like to hear each other’s stories at the event?
  - How does it impact how you may see or think about people who you come across in the hospital?
  - How has it made you think about other stories you have, if at all?
- How do you think storytelling events like BIDMC Stories could help build a sense of community and connection at BIDMC?
  - What would help this sense of community and connection to persist?
- How do you think storytelling events can help in mitigating burnout and promoting wellness for people who attend the events, if at all?
- Do you have any suggestions for ways that we could improve this event in the future?
- Would you be interested in being involved with BIDMC Stories in the future?
  - Would you be interested in helping to plan future storytelling events?
  - Would you be interested in keeping in touch with your fellow storytellers? If so, in what way?
